# Neurofunctional Changes Following Multimodal Rehabilitation Treatment in Patients with Cervical Spondylosis: An Exploratory Electrophysiological Study

**DOI:** 10.3390/diagnostics16152336

**Published:** 2026-07-25

**Authors:** Andreea Ancuta Talinga, Roxana Ramona Onofrei, Veronica Aurelia Romanescu, Ada-Maria Codreanu, Marius-Zoltan Rezumes, Alexandra Laura Mederle, Liliana Catan, Dan-Andrei Korodi, Oana Suciu, Alexandru Caraba, Claudia Borza

**Affiliations:** 1 Doctoral School, “Victor Babeș” University of Medicine and Pharmacy Timișoara, Eftimie Murgu Sq. No. 2, 300041 Timişoara, Romania; andreea.vataman@umft.ro (A.A.T.); veronica.romanescu-pesecan@umft.ro (V.A.R.); ada.codreanu@umft.ro (A.-M.C.); marius.rezumes@umft.ro (M.-Z.R.); alexandra.mederle@umft.ro (A.L.M.); 2 Center for Translational Research and Systems Medicine, “Victor Babeș” University of Medicine and Pharmacy Timișoara, Eftimie Murgu Sq. No. 2, 300041 Timişoara, Romania; 3 Department of Rehabilitation, Physical Medicine and Rheumatology, “Victor Babeș” University of Medicine and Pharmacy Timișoara, Eftimie Murgu Sq. No. 2, 300041 Timișoara, Romania; oanasuciu78@umft.ro; 4 Department of Rehabilitation, Physical Medicine and Rheumatology, Research Center for Assessment of Human Motion, Functionality and Disability, “Victor Babeș” University of Medicine and Pharmacy Timișoara, Eftimie Murgu Sq. No. 2, 300041 Timișoara, Romania; catan.liliana@umft.ro; 5 Department of Medicine, Faculty of Medicine, “Vasile Goldiș” Western University of Arad, L. Rebreanu St. 86, 310048 Arad, Romania; korodi.andrei@uvvg.ro; 6 Department of Internal Medicine, Diabetes and Systemic Rheumatology, “Victor Babeș” University of Medicine and Pharmacy Timișoara, Eftimie Murgu Sq. No. 2, 300041 Timișoara, Romania; caraba.alexandru@umft.ro; 7 Department of Functional Sciences—Pathophysiology, Center for Translational Research and Systems Medicine, “Victor Babeș” University of Medicine and Pharmacy Timișoara, Eftimie Murgu Sq. No. 2, 300041 Timişoara, Romania; borza.claudia@umft.ro

**Keywords:** cervical spondylosis, median nerve, abductor pollicis brevis, nerve conduction studies, semiquantitative surface electromyography, electrodiagnostic assessment, multimodal rehabilitation treatment, neuromuscular function, rehabilitation, pain assessment

## Abstract

**Background/Objectives**: This study aimed to assess nerve conduction studies (NCS) parameters of the median nerve and abductor pollicis brevis (APB) muscle activity in patients with cervical spondylosis. Additionally, we evaluated longitudinal changes in these parameters at six months following a multimodal rehabilitation treatment. **Methods**: This quasiexperimental, nonrandomized study included 41 patients with cervical spondylosis and 25 healthy controls. Median nerve conduction studies and semiquantitative surface electromyography (sEMG) of the APB muscle were assessed using a two-channel Neuro-MEP-Micro system (version 2009, Neurosoft Ltd.). The evaluations were performed at baseline in both groups and repeated after six months in the patient group. Pain intensity was assessed using the Visual Analog Scale (VAS). **Results**: Compared with controls, patients showed reduced compound muscle action potential (CMAP) amplitude (Cohen’s *d* = 1.53–1.83), slower motor conduction velocity (*r* = 0.91–0.95), and impaired sEMG amplitude and recruitment pattern of the APB muscle (all adjusted *p* < 0.001). After application of multimodal rehabilitation treatment, VAS scores decreased significantly (adjusted *p* < 0.001), and favorable changes in NCS and sEMG parameters were observed, with moderate to large effect sizes (Cohen’s *d* = 0.56–0.82; *r* = 0.52–0.86; all adjusted *p* ≤ 0.006). CMAP amplitude correlated positively with sEMG amplitude and recruitment pattern (ρ = 0.483–0.632, all adjusted *p* ≤ 0.001). **Conclusions**: Multimodal rehabilitation treatment may be associated with clinical and electrophysiological improvements, suggesting the complementary value of NCS and sEMG for the evaluation and follow-up of patients with cervical spondylosis. However, because of the nonrandomized uncontrolled design and the absence of longitudinal follow-up in controls, causal treatment effects cannot be inferred.

## 1. Introduction

Cervical degenerative processes involve progressive changes in the intervertebral disks, uncovertebral and facet joints, and osteophyte formation. These alterations may lead to reduced disk height, segmental instability, and foraminal or spinal canal stenosis, with possible compression of adjacent neural structures [1,2]. Clinically, cervical spondylosis is associated with neck pain and, in some patients, cervicobrachial radiculopathy, which may affect upper limb neuromuscular function and fine motor performance [3,4].

In this context, electrodiagnostic investigations provide information regarding the functional integrity of peripheral nerves and the neuromuscular system. Nerve conduction studies (NCS) allow the assessment of latencies, amplitudes, and motor conduction velocities, and are useful for identifying peripheral nerve involvement and differentiating radiculopathy from other neuropathies. Electromyography further contributes to the characterization of neuromuscular impairment [5,6].

The median nerve and the APB muscle were selected because median motor NCS are performed by recording the CMAP from the APB. This approach provides reproducible measurements of distal motor latency, CMAP amplitude, and motor conduction velocity [7,8,9]. The purpose of the present study was not to electrophysiologically localize a specific cervical root lesion, but to evaluate distal median motor function and semiquantitative APB muscle activity in patients with cervical spondylosis. Accordingly, the electrophysiological findings should be interpreted as exploratory measures of distal upper limb neurofunctional involvement.

However, because median nerve conduction abnormalities may also occur in peripheral entrapment neuropathies, such as carpal tunnel syndrome (CTS) or metabolic neuropathies, the electrophysiological findings of parameters assessed in this study were exploratory indicators of distal upper limb neurofunctional involvement [7,10,11].

Rehabilitation treatment aims to reduce pain and improve cervical mobility and upper limb function. A multimodal approach in rehabilitation may contribute to symptom relief and reduction in disability [3,12,13,14]. Neurofunctional assessment may also contribute to monitoring rehabilitation changes and functional prognosis in patients with cervical spondylosis. In the present study, we used the following procedures in the rehabilitation treatment protocol: balneotherapy, electrotherapy, thermotherapy, and kinetotherapy.

The aim of this study was to evaluate distal median nerve conduction parameters and APB muscle activity in patients with cervical spondylosis using NCS and semiquantitative sEMG, and to describe their longitudinal changes at six months following multimodal rehabilitation treatment in the patient group.

The working hypothesis was that patients with cervical spondylosis would present significant electrodiagnostic alterations compared to healthy subjects, and that NCS and semiquantitative sEMG parameters may show favorable changes in follow-up.

## 2. Materials and Methods

### 2.1. Study Design

This study was conducted in a rehabilitation clinic in Timiș County and followed a quasi-experimental, non-randomized design with longitudinal assessment of the patient group. The intervention consisted of a multimodal rehabilitation treatment applied to all enrolled patients, without random allocation or an untreated control group. This manuscript was prepared and reported in accordance with the Strengthening the Reporting of Observational Studies in Epidemiology (STROBE) Statement (Table A1) [15].

The primary outcome was the evaluation of electrophysiological function using median nerve conduction and semiquantitative sEMG parameters of the APB muscle. Secondary outcomes included pain intensity assessed by the VAS scale and exploratory analyses of the associations between electrophysiological findings and MRI abnormalities.

The sample included 41 patients diagnosed with cervical spondylosis between March 2023 and August 2025, as well as 25 healthy subjects who served as a control group. The sample size was determined by the number of eligible patients consecutively presenting to the clinic during the study period and consenting to participate.

Assessments were performed at baseline and six months after completion of the rehabilitation treatment to evaluate changes in clinical and electrophysiological parameters. Control subjects were evaluated only once to provide reference values.

This research was conducted in accordance with the Declaration of Helsinki and approved by the Ethics Committee of the “Victor Babeș” University of Medicine and Pharmacy Timișoara, Romania (protocol no. 28/10 January 2022). Written informed consent was obtained from all participants before inclusion.

### 2.2. Patient Group

The study group consisted of 41 adult patients with cervical spondylosis diagnosed based on imaging and clinical examination. Patients had a cervical MRI or radiography performed prior to presentation, and results revealed degenerative disk disease associated with protrusion, disk herniation, and spinal canal stenosis, conditions frequently associated with neck pain and radicular symptoms affecting the upper limb [16,17,18]. MRI findings were numerically coded as follows: 1—disk protrusion, 2—disk herniation, and 3—spinal canal stenosis for exploratory subgroup analysis of pain outcomes in statistical analysis and should not be interpreted as a validation ordinal severity scale. In our research, patients with cervical spondylosis were analyzed as a single study population and were not stratified according to the anatomical level of cervical involvement.

Demographic and clinical data were collected, including age, height, body weight, and occupational status. Comorbidities with potential impact on neuromuscular function, such as hypertension and diabetes mellitus, were also recorded. In the patient group, additional disease-related variables were documented, including symptom duration expressed in months and the presence of radiculopathy. The prior use of nonsteroidal anti-inflammatory drugs (NSAIDs) and weak opioids was also recorded.

Pain intensity was assessed using the Visual Analog Scale (VAS), a validated and widely used tool for pain quantification in musculoskeletal disorders [19,20]. Evaluations were performed at baseline and six months after rehabilitation treatment.

Inclusion criteria were confirmed diagnosis of cervical spondylosis, presence of neck pain with or without cervicobrachial radiculopathy, availability for both baseline and 6-month follow-up assessments, and provision of informed consent. Exclusion criteria included peripheral neuropathies of other etiologies (carpal tunnel syndrome, radial nerve palsy, cubital tunnel syndrome, diabetic neuropathy), prior cervical spine surgery, cervical trauma or spinal cord injury, primary neuromuscular disorders, and severe systemic diseases that could influence NCS parameters.

### 2.3. Control Group

The control group consisted of 25 healthy volunteers recruited from the general population with no history of cervical spondylosis, chronic neck pain, radicular symptoms, or known neurological disorders. All control subjects underwent a single baseline evaluation to establish reference values for the parameters investigated. Demographic data, including age, height, weight, and occupational status, were recorded based on the anamnesis. No matching between groups was performed based on age, sex, or anthropometric characteristics; therefore, these variables were considered confounders in the statistical analysis.

### 2.4. Electrophysiological Assessment

Neurofunctional evaluation included NCS and sEMG of the median nerve and APB muscle, bilaterally. Examinations were performed using a two-channel Neuro-MEP-Micro system (version 2009, Neurosoft Ltd., Ivanovo, Russia) [21], in a room with an ambient temperature of 22 °C, in order to reduce the potential influence on nerve conduction parameters.

**Figure 1:**
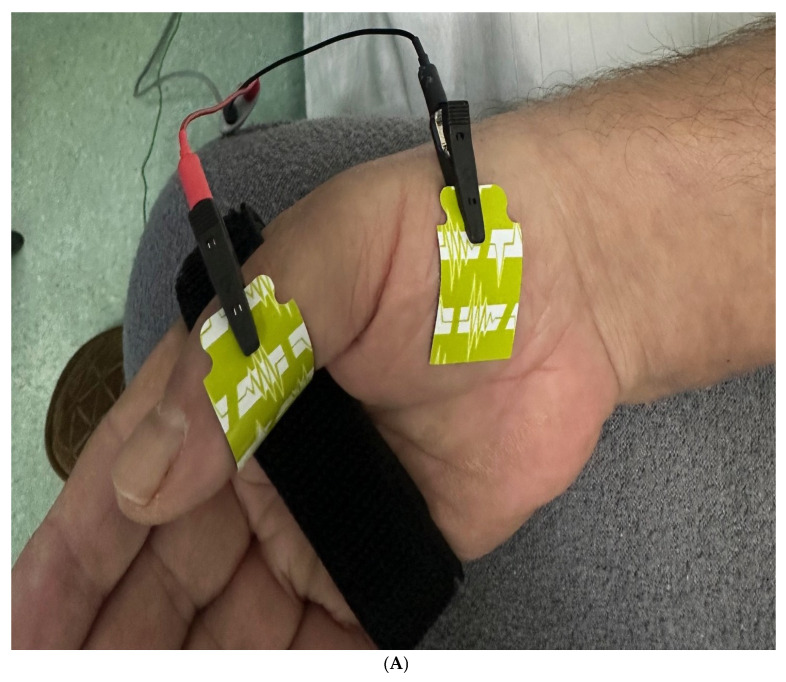

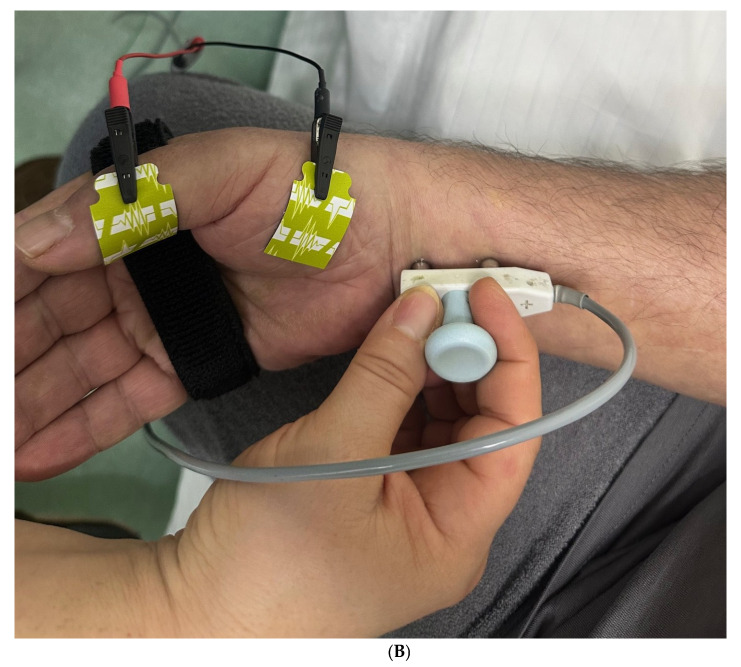
Electrodes placement for the median nerve (**A**); stimulation point (**B**).

Participants were examined in a seated position, with the upper limbs relaxed and supported, to avoid involuntary muscle activity. Surface adhesive electrodes were used for recording and were placed according to standardized electrodiagnostic techniques [7]. The active electrode was placed over the thenar eminence, while the reference electrode was positioned distally on the thumb (Figure 1A). Stimulation was applied at the wrist, between the tendons of the flexor carpi radialis and palmaris longus (Figure 1B).

We assessed the following NCS parameters: distal latency (ms), residual latency (ms), compound muscle action potential (CMAP) amplitude (mV), and motor conduction velocity (m/s). These parameters were used in studies to evaluate nerve fiber integrity and to differentiate between demyelinating and axonal involvement [7,8,22].

At the 6-month follow-up, the same measurements were repeated on the same muscle and nerve evaluated at baseline for each patient, while the control group was examined only once at baseline. To reduce interobserver variability, all assessments were performed by the same examiner. Since electrophysiological parameters may be influenced by demographic and metabolic factors [9], and the groups were not strictly matched, multivariate linear regression models were used to assess the independent effect of group.

#### Surface Electromyography (sEMG)

**Figure 2:**
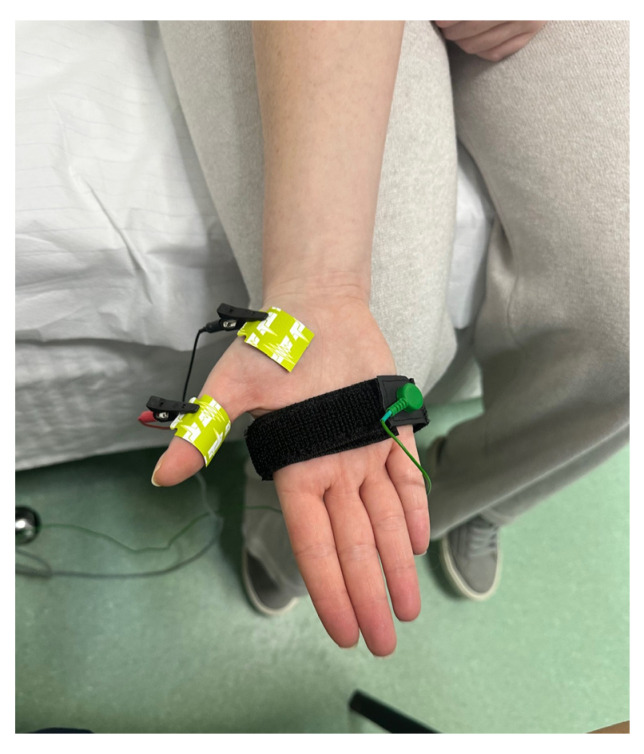
Electrodes placement for sEMG of APB muscle.

The same 2-channel Neuro-MEP-Micro system (version 2009, Neurosoft Ltd.) was used to assess APB muscle activity, and surface adhesive electrodes were placed over the muscle belly, parallel to muscle fiber orientation (Figure 2), to optimize signal acquisition and minimize interference from adjacent muscles [23,24,25].

**Figure 3:**
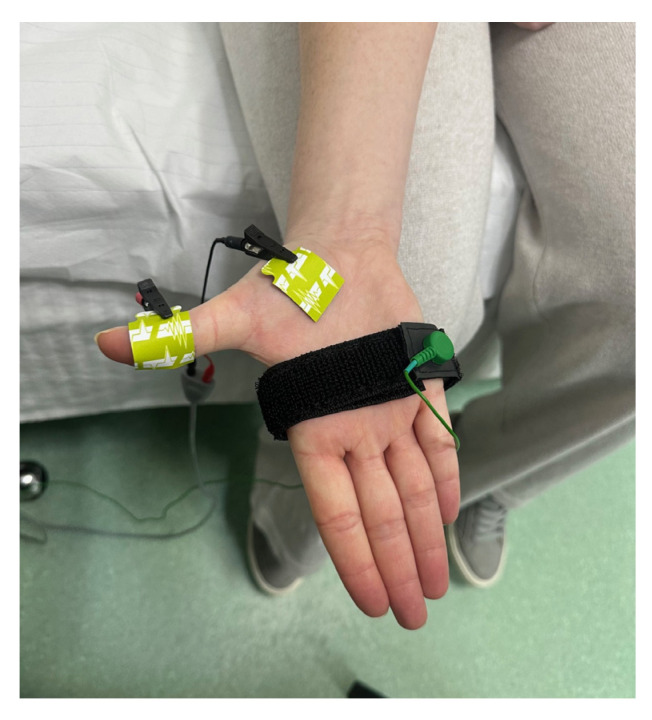
Maximal voluntary thumb abduction.

The examination was performed with the upper limb in a relaxed and supported position. Participants were instructed to perform maximal voluntary thumb abduction upon the examiner’s command, avoiding compensatory movements (Figure 3). They did two test contractions before recording.

The examined parameters in this study were semiquantitative sEMG amplitude, recruitment pattern, and duration. Because the assessment was designed as a semiquantitative evaluation, quantitative EMG parameters, including signal filtering characteristics, root mean square (RMS) analysis, normalization procedures, and reliability testing, were not recorded. The Neuro-MEP-Micro system automatically generated standardized semiquantitative descriptive categories for the examined parameters, which were displayed in the Neurosoft software application (Neuro-MEP.NET Omega version 4.2.5.9; Neurosoft LLC, Ivanovo, Russia). These software-generated categories were converted by the investigator into an ordinal 0–5 scale solely for statistical analysis, where 5 represented normal activity, 4 near normal activity, 3 mildly reduced activity, 2 moderately reduced activity, 1 severely reduced activity, and 0 as absence of organized motor activity. All assessments were performed by the same examiner, who was not blinded, at baseline in both groups and repeated after 6 months only in the patient group.

### 2.5. Rehabilitation Treatment Protocol

Patients in the study group underwent a rehabilitation treatment with a duration of 14 consecutive days, administered in the clinic under medical supervision. The protocol had a multimodal structure and aimed to reduce pain, improve cervical mobility, and optimize upper limb neuromuscular function. This included balneotherapy, electrotherapy, thermotherapy, and kinetotherapy applied in the same sequence for all patients (Table 1 and Table 2).

Balneotherapy consisted of carbonated mineral water baths at a temperature of 33–34 °C with a duration of 20 min per session, followed by a rest period of approximately one hour to allow autonomic stabilization according to this study [26,27]. The mineral water used in this study was a mixture of carbonated, bicarbonate, magnesium–calcium, chlorinated, brominated, and calcium-rich waters. Its chemical composition included CO_2_ (1443.2 mg/L), Cl (579.9 mg/L), bicarbonate (2025.2 mg/L), Mg (110.2 mg/L), Fe (3 mg/L), Na (685.5 mg/L), K (19 mg/L), Ca (206.8 mg/L), bromine (0.3 mg/L), and iodine (0.1 mg/L), with a total mineralization of 3743.7 mg/L, according to the water analysis certificate no. 46/19.01.2023 issued by the National Institute of Rehabilitation, Physical Medicine and Balneology, Bucharest, Romania.

Electrotherapy was applied using transcutaneous electrical nerve stimulation (TENS) at the cervical paravertebral level. Electrodes were positioned in one or two longitudinal circuits, depending on the extent of the painful area. Stimulation intensity was adjusted to produce a comfortable tingling sensation, according to patient tolerance. Each session lasted approximately 20 min [28].

Ultrasound therapy was applied to the cervical paravertebral region using a frequency of 1 MHz and an intensity of 0.5 W/cm^2^ in continuous mode. The duration was determined according to the treated surface area, at approximately one minute per 5 cm^2^ [29].

Thermotherapy consisted of paraffin and wax applications in the form of a “cervical cape” covering the posterior cervical region and shoulders. The temperature ranged between 38 and 40 °C, and the duration was 20 min, followed by a one-hour rest period [28].

Kinetotherapy represented the main active component of the rehabilitation program and was focused on improving cervical mobility, enhancing neuromuscular control, and restoring functional performance of the upper limb. The program included active cervical mobilization exercises, such as flexion, extension, rotation, and lateral inclination, performed in a controlled manner within pain-free limits (2 sets of 8–10 repetitions per direction), followed by isometric exercises targeting cervical musculature, with contractions maintained for 6 s.

Cervical stabilization exercises were also performed, particularly targeting deep cervical flexors through chin tuck maneuvers, executed in 2 sets of 6–8 repetitions with a 5–10 s hold. Additional exercises included scapulohumeral stabilization and progressive resistance training, as well as stretching exercises for cervical and shoulder girdle muscles. Exercise intensity and range of motion were gradually increased based on patient tolerance, particularly in individuals with cervical radiculopathy, to avoid exacerbation of symptoms. They were performed at a slow to moderate velocity and discontinued if they provoked a marked increase in pain or neurological symptoms. Sessions lasted approximately 40 min and were performed daily for 14 days under direct supervision to ensure proper execution.

After completion of the supervised rehabilitation program, patients were instructed to continue the kinetotherapy at home, twice a week with 30 min per session. The advised program included cervical mobility exercises, isometric cervical exercises, and stretching. Adherence to the program at home was not monitored during the six-month period. A detailed description of the rehabilitation intervention according to the TIDieR checklist is provided in Appendix B [30].

### 2.6. Statistical Analysis

Statistical analysis was performed using MedCalc version 23.4.5 (MedCalc Software Ltd., Ostend, Belgium). The distribution of continuous variables was assessed using the Shapiro–Wilk test. Normally distributed data were expressed as mean ± standard deviation, while abnormally distributed data were reported as median and interquartile range. Because no a priori sample size calculation was performed, a post hoc sensitivity analysis was conducted in G*Power version 3.1.9.4 for the multivariable linear regression models. Assuming α = 0.05, statistical power = 0.80, a total sample size of 66 participants, and six predictors (group, age, sex, BMI, hypertension, and diabetes mellitus), G*Power computed a detectable effect size of f^2^ = 0.229, corresponding to a moderate to large effect size according to Cohen’s classification [31].

Standardized baseline differences were calculated to assess comparability between patients and controls. We used the following formulas:

for continuous variables

$$SMD=\left(\bar{X}patients-\bar{X}controls\right)/\surd \left[\frac{SDpatients^{2}+SDcontrols^{2}}{2}\right]$$

for categorical variables

$$SMD=(Ppatients-Pcontrols)\;/\;\surd \left\{\frac{\left[Ppatients\left(1-Ppatients\right)+Pcontrols\left(1-Pcontrols\right)\right]}{2}\right\}$$

Absolute values were reported, with values ≥ 0.10 considered indicative of baseline imbalance [32].

Comparisons between groups were performed using Student’s *t*-test or Mann–Whitney U test, depending on data distribution. Longitudinal comparisons within the patient group were conducted using the paired *t*-test or Wilcoxon signed-rank test. For comparisons across MRI subgroups, the Kruskal–Wallis test was applied, followed by Conover post hoc analysis. Associations between clinical variables and electrophysiological parameters were assessed using Spearman’s correlation coefficient.

Multivariate linear regression was performed to adjust for confounders in cross-sectional analyses. Regression coefficients (B) with 95% confidence intervals were reported. Model assumptions were verified through residual analysis and multicollinearity assessment using the variance inflation factor (VIF). *p*-values were adjusted using the Holm–Bonferroni method to control for type I error due to multiple testing. Statistical significance was set at *p* < 0.05.

As an exploratory analysis, distribution-based estimates of the minimal clinically important difference (MCID) were calculated for each NCS parameter using one-half of the pooled standard deviation (0.5 × pooled SD) of baseline and follow-up measurements, following the distribution-based approach applied by Ashoori et al. [33]. The pooled standard deviation was calculated as

$$SD_{pooled}=\sqrt{\frac{SD_{T0}^{2}+SD_{T1}^{2}}{2}}$$

The estimated MCID was then calculated as

$$MCID=0.5\times SD_{pooled}$$

## 3. Results

### 3.1. Cross-Sectional Analysis

#### 3.1.1. Demographic and Clinical Characteristics

The study included 66 participants, comprising 41 patients with cervical spondylosis (62.1%) and 25 healthy controls (37.9%). Standardized baseline differences confirmed imbalance between groups, particularly for age, height, BMI, hypertension, and diabetes mellitus. However, after Holm–Bonferroni correction, only age remained statistically significant. These variables were considered potential confounders in the interpretation of cross-sectional comparisons and were included in adjusted regression analyses (Table 3).

#### 3.1.2. Clinical Characteristics of the Patient Group

In this study, the median value of symptom duration was 6 months [IQR 3.75–9] (Table 4). Cervical radiculopathy was present in 80.5% of patients and was associated with a significantly longer symptom duration (adjusted *p* = 0.003).

Regarding prior treatment, 65.9% of patients used NSAIDs and 61.0% used weak opioids. Opioid use was associated with longer symptom duration (adjusted *p* < 0.001), whereas NSAID use showed no significant association (adjusted *p* = 0.392).

MRI was performed in 68.3% of patients, while 48.8% underwent cervical radiography. Among patients evaluated by MRI, the most frequent findings were degenerative disk disease and disk herniation (each 42.9%), followed by spinal stenosis (14.3%). No significant differences in symptom duration were observed across imaging subgroups (adjusted *p* = 0.196).

#### 3.1.3. Changes in NCS Parameters of the Median Nerve

Cross-sectional analysis was associated with significant differences in all nerve conduction parameters of the median nerve, bilaterally, between patients and controls. Distal motor latency was prolonged bilaterally in patients (*p* < 0.001 after Holm–Bonferroni correction) with a median increase of approximately 1.0–1.1 ms; CMAP amplitude was reduced in the patient group (*p* < 0.001) with large effect sizes (Cohen’s d ≥ 1.53) and motor conduction velocity was significantly decreased bilaterally in patients (*p* ≤ 0.002 after adjustment), with median reductions of approximately 3.4–5.0 m/s (Table 5).

#### 3.1.4. Multivariable Linear Regression Analyses Adjusted for Confounders

Multivariable linear regression analyses adjusted for age, sex, BMI, and comorbidities showed persistent differences between the groups in all NCS parameters. Patients showed increased distal latency (right: B = 1.15, *p* = 0.011; left: B = 1.10, *p* < 0.001) and residual latency (right: B = 1.13, *p* = 0.006; left: B = 0.98, *p* < 0.001). CMAP amplitude was reduced bilaterally (right: B = −4.14, *p* < 0.001; left: B = −3.38, *p* < 0.001), while motor conduction velocity was significantly decreased (right: B = −4.00, *p* = 0.016; left: B = −4.80, *p* = 0.004). Regression diagnostics did not identify deviations from model assumptions, with VIF values below 3.8.

#### 3.1.5. Surface Electromyography of the Abductor Pollicis Brevis Muscle

Nonparametric analysis showed a significant reduction in semiquantitative sEMG amplitude in patients with cervical spondylosis compared with healthy controls for the APB muscle bilaterally (*p* < 0.001), with large effect sizes. Recruitment pattern was also significantly reduced in patients on both sides (Table 6). In contrast, sEMG signal duration was normal in both groups and showed no variability, resulting in a ceiling effect that limited its discriminatory value.

### 3.2. Longitudinal Analysis

#### 3.2.1. Pain Outcomes After Multimodal Rehabilitation Treatment

VAS scores decreased significantly following the multimodal rehabilitation treatment, suggesting improvements in patient pain status (adjusted *p* < 0.001). The median reduction estimated using the Hodges–Lehmann method was 2.5 points, exceeding the commonly reported minimal clinically important difference (MCID) for chronic musculoskeletal pain [34,35]. The change in pain intensity (ΔVAS) was calculated as baseline VAS scores minus follow-up scores, with higher positive values indicating greater pain reduction. Correlation analysis did not reveal significant associations between ΔVAS and clinical or demographic variables, including age, symptom duration, BMI, hypertension, and diabetes (Table 7).

No significant differences in ΔVAS were observed according to the presence of radiculopathy (*p* = 0.594). In the exploratory MRI subgroup analysis, ΔVAS differed significantly across imaging categories, and we performed a post hoc Conover analysis where patients with disk protrusion showed a greater reduction in pain compared with those with disk herniation (*p* = 0.031), while no significant differences were observed for spinal stenosis associated with cervical spondylosis.

#### 3.2.2. Longitudinal Analysis of NCS Parameters of the Median Nerve

A 6-month longitudinal analysis was performed in the patient group to evaluate changes in median nerve conduction parameters between baseline and follow-up. Distal latency decreased significantly bilaterally (adjusted *p* < 0.001), with large effect sizes. CMAP amplitude increased significantly on both sides (right: adjusted *p* < 0.001; left: adjusted *p* = 0.004), with moderate to large effect sizes. MCV also showed a significant increase at 6 months bilaterally (right: adjusted *p* < 0.001; left: adjusted *p* = 0.006) (Table 8). Exploratory MCID estimates were calculated for the longitudinal NCS parameters. Although all parameters showed statistically significant changes, the observed mean changes remained below the corresponding estimated MCID values.

#### 3.2.3. Longitudinal Semiquantitative sEMG Analysis of the Abductor Pollicis Brevis Muscle

In longitudinal semiquantitative sEMG analysis, we observed significant favorable changes in APB amplitude and recruitment scores bilaterally, following multimodal rehabilitation treatment (Table 9).

### 3.3. Exploratory Correlation Analyses

Spearman correlation analysis was performed to evaluate the association between CMAP amplitude and semiquantitative sEMG parameters of the APB muscle. For the right side, CMAP amplitude showed a positive correlation with sEMG amplitude (ρ = 0.632, *p* < 0.001), while the association with recruitment pattern was similar (ρ = 0.626, *p* < 0.001). For the left side, correlations were moderate but remained statistically significant, both for sEMG amplitude (ρ = 0.483, *p* = 0.001) and recruitment pattern (ρ = 0.518, *p* < 0.001).

In our study, no significant correlations were identified between ΔVAS and ΔCMAP amplitude (right: ρ = −0.093, *p* = 0.596; left: ρ = 0.018, *p* = 0.919); regarding ΔVAS and changes in APB recruitment pattern, we did not find significant correlations either (right: ρ = 0.052, *p* = 0.819; left: ρ = 0.115, *p* = 0.621).

## 4. Discussion

In the present study, cervical spondylosis was evaluated as a single clinical entity without stratification according to the anatomical level of cervical involvement. Therefore, our objective was to assess distal median motor function using motor nerve conduction studies and semiquantitative APB muscle activity. The observed electrophysiological findings should not be interpreted as evidence of a specific cervical root lesion because NCS alone cannot localize cervical radiculopathy, and needle EMG remains the principal electrodiagnostic method for confirming the diagnosis [36,37].

The results of our study showed differences in median nerve conduction parameters and semiquantitative APB muscle activity between patients with cervical spondylosis and healthy controls, characterized by prolonged latencies, reduced CMAP amplitude, and slower motor conduction velocity (all adjusted *p* < 0.001). These electrophysiological results should be interpreted as exploratory findings of distal upper limb neurofunctional involvement because similar abnormalities may also occur in peripheral entrapment neuropathies, including CTS or metabolic neuropathies [6].

The cross-sectional analysis revealed a reduction in CMAP amplitude (*p* < 0.001) and a slowing of motor conduction velocity (*p* ≤ 0.002). These results should be interpreted cautiously, since NCS findings in radiculopathy may be normal or mildly abnormal [6]. Although patients had imaging evaluation confirming cervical spondylosis, the observed changes in the NCS parameters of the median nerve should not be considered specific, since subclinical peripheral nerve entrapment or metabolic influences cannot be completely excluded [38]. Therefore, the observed electrophysiological results presented in this study are exploratory.

Previous studies have shown that patients with chronic neck pain exhibit altered muscle activation patterns, although most investigations focus on cervical and paraspinal muscles rather than distal muscles such as the APB [39,40]. Mendez-Rebolledo et al. demonstrated that both experimental and clinical pain can modify the spatial distribution of muscle activity [41], while narrative reviews suggest that electromyographic changes may reflect neuromuscular adaptations associated with chronic neck pain [39].

The longitudinal analysis showed changes in latencies, CMAP amplitude, and motor conduction velocity between baseline and the six-month follow-up (all *p* ≤ 0.006). These results suggest that conservative treatment may be associated with symptom relief and changes in neuromuscular function; however, because of the absence of randomization and a longitudinal analysis of the control group, these changes cannot be attributed exclusively to the intervention and should be considered exploratory. Other studies have also described improvements in pain and functional outcomes after multimodal rehabilitation programs, which is broadly consistent with the approach used in our study [42,43,44,45].

The mechanisms underlying these electrophysiological improvements are probably multifactorial. A systematic review and meta-analysis by Parker et al., including 43 studies (1009 patients with chronic pain and 658 controls), demonstrated that chronic pain is associated with altered corticospinal excitability and reduced intracortical inhibition, suggesting that pain itself may contribute to impaired motor output [46]. In addition, Sanderson et al. reviewed 61 studies and reported that chronic musculoskeletal pain may alter motor unit behavior and recruitment patterns, whereas improvements in pain may facilitate more efficient neuromuscular activation [47]. More recently, a systematic review and meta-analysis by Dirito et al., including 14 studies in patients with chronic neck pain, concluded that exercise interventions induce neuromuscular adaptations measurable by electromyography and improve motor control [48]. Although these mechanisms were not directly investigated in the present study, they may partially explain the observed increases in CMAP amplitude and the improvement in semiquantitative recruitment pattern following rehabilitation.

In the cross-sectional analysis of the exploratory semiquantitative sEMG results, patients showed reduced amplitude and altered recruitment patterns at baseline, whereas higher amplitude scores and improved recruitment patterns were observed at the follow-up (all adjusted *p* ≤ 0.006). In a recent study, De Zoete et al. suggested that contemporary exercise approaches should target not only pain reduction but also restoration of motor control [49]. Exercise-based interventions, including cervical and scapular stabilization programs, have shown benefits in pain and functional outcomes in patients with chronic neck pain [50,51,52].

Recent studies showed that cervical degenerative MRI findings do not always correlate closely with pain intensity or disability [53,54]. In our previous research on patients with lumbar spondylosis associated with radiculopathy, we also observed that NCS may provide useful functional information regarding peripheral nerve damage and may complement clinical and imaging assessment [55]. In this study, MRI findings were retained for exploratory subgroup analysis of pain outcomes, and we observed that patients with cervical spondylosis associated with disk protrusion showed greater VAS score reduction compared with those with degenerative disk herniation (*p* = 0.031).

Overall, from a clinical perspective, these exploratory results suggest the potential role of electrophysiological assessment as a complementary tool in the rehabilitation monitoring of patients with cervical spondylosis. Integrated neurofunctional assessment may improve the characterization of neuromuscular dysfunction and provide objective measures for monitoring changes in patients undergoing rehabilitation. The present study is part of a broader doctoral research project assessing neurofunctional parameters in cervical spondylosis, and we report our exploratory findings regarding median nerve conduction and APB muscle activity, while results concerning other peripheral nerves, respectively, the cubital and radial nerves, will be reported in future studies.

### 4.1. Clinical Relevance of the Findings

Although the present study demonstrated statistically significant improvements in pain and electrophysiological parameters following multimodal rehabilitation, the clinical relevance of these outcomes should be interpreted separately. The median VAS reduction of 2.5 points exceeded the commonly reported MCID for chronic musculoskeletal pain, suggesting that the observed changes were not only statistically significant but also clinically meaningful. Previous studies and the IMMPACT recommendations indicate that a reduction of approximately two points or 30% represents a clinically meaningful improvement in chronic pain outcomes [34,35].

In contrast, no validated anchor-based MCID values have been established for motor NCS or semiquantitative sEMG parameters in patients with cervical spondylosis.

In a recent study, Ashoori et al. calculated study-specific MCID values for tibial nerve distal latency, CMAP amplitude, motor conduction velocity, and F-wave latency in patients with diabetic peripheral neuropathy using one-half of the pooled standard deviation [33]. However, the authors emphasized that these thresholds were sample-dependent, statistically derived, and should not be considered universally applicable or interpreted as patient-perceived clinical benefit.

Following their approach, in our study we calculated exploratory MCID values for all NCS parameters. The MCID values ranged from 0.457 to 0.900 ms for distal and residual latencies, 1.225–1.422 mV for CMAP amplitude, and 3.295–3.414 m/s for MCV. Despite reaching statistical significance, the observed mean changes in all NCS parameters remained below the corresponding exploratory MCID values, indicating that their magnitude did not reach the estimated threshold for clinically meaningful change. Quantitative sEMG studies have reported indices such as the standard error of measurement (SEM), minimal detectable change (MDC), and test–retest reliability [56]; however, no validated MCID has been established for the semiquantitative sEMG method used in our study. Therefore, the clinical relevance of the semiquantitative sEMG findings could not be interpreted using MCID criteria.

### 4.2. Study Limitations

We acknowledge several methodological limitations. First, the study had a quasi-experimental design without randomization or a treated control group. Since all patients received the rehabilitation treatment and controls were evaluated only once at baseline, it cannot be determined whether the observed improvements were related to the rehabilitation itself, the natural course of the disease, increased attention associated with study participation, including the Hawthorne effect, or regression toward the mean. Therefore, the improvements observed at the six-month follow-up should be interpreted as associations occurring after rehabilitation rather than definitive treatment effects.

Second, the subjects of the two groups were not matched for age, sex, BMI, or height, which may have influenced electrophysiological parameters despite statistical adjustment. The study was not registered on a trial registry.

Another limitation is that sensory nerve conduction studies and needle electromyography were not performed; therefore, electrophysiological localization of cervical root involvement could not be established. The study also used a semiquantitative ordinal scale for sEMG, which, although clinically practical, is less sensitive than standardized quantitative electromyographic methods [57,58]. Because raw EMG signals were not retained during data acquisition, the study did not include detailed quantitative sEMG signal processing parameters, and the inter-rater and intra-rater reliability of the semiquantitative sEMG scoring system were not assessed. Limb skin temperature was not systematically monitored during electrophysiological assessments.

Furthermore, the multimodal rehabilitation treatment included therapeutic procedures administered simultaneously, making it impossible to determine the specific contribution of each modality in the observed changes. In addition, medication use, cointerventions, and adherence to a physical exercise program at home were not monitored during the six months.

Finally, the sample size, n = 66 subjects, including 41 patients and 25 healthy controls, was relatively modest, which may limit the generalizability of the findings. Larger controlled longitudinal studies are needed to confirm these exploratory results.

## 5. Conclusions

Patients with cervical spondylosis showed altered median nerve conduction parameters and impaired semiquantitative APB muscle activity compared with healthy subjects. Following multimodal rehabilitation treatment, favorable longitudinal changes were observed in both NCS and semiquantitative sEMG parameters, together with pain reduction. The combined neurofunctional assessment of median nerve conduction and APB muscle activity using NCS and semiquantitative sEMG may be useful for monitoring neurofunctional changes in patients with cervical spondylosis undergoing rehabilitation treatment.

However, because of the nonrandomized design, unmatched groups, and absence of a longitudinal follow-up in controls, the observed changes should be interpreted as exploratory findings rather than evidence of causal treatment effects.

## Figures and Tables

**Table 1:** Physiotherapy procedures.

Component	Intervention Details	Parameters	Duration/Frequency
Balneotherapy	CO_2_ mineral water bath	33–34 °C	20 min/session + 1 h rest
TENS	Cervical paravertebral application	Intensity adjusted to patient tolerance	20 min/session
Ultrasound Therapy	Cervical paravertebral region	1 MHz, 0.5 w/cm^2^, continuous mode	~1 min/5 cm^2^
Thermotherapy	Paraffin–wax “cervical cape” application	38–40 °C	20 min/session + 1 h rest

**Abbreviations**: TENS, transcutaneous electrical nerve stimulation. All procedures were performed under medical supervision and adjusted according to patient tolerance.

**Table 2:** Kinetotherapy protocol.

Component	Intervention Details	Parameters	Duration/Frequency
Kinetotherapy	Active mobilization (Flexion, extension, rotation, lateral inclination)	2 × 8–10 reps	Daily
	Isometric exercises	2 × 8–10 reps, 6 s hold	Daily
	Deep cervical flexors (chin tuck)	2 × 6–8 reps, 5–10 s hold	Daily
	scapulohumeral stabilization + stretching for cervical and shoulder girdle muscles	Progressive	Daily

**Table 3:** Demographic and clinical characteristics of the study groups.

Variable	Patients (*n* = 41)	Controls (*n* = 25)	Adjusted *p*	Standardized Difference
Age (years), mean ± SD	57.02 ± 11.74	45.24 ± 13.21	**<0.001**	**0.942**
Weight (kg), mean ± SD	72.44 ± 14.02	72.52 ± 17.05	0.983	0.005
Height (cm), mean ± SD	163.61 ± 9.12	170.16 ± 11.42	0.091	**0.633**
BMI (kg/m^2^), mean ± SD	26.96 ± 4.10	24.75 ± 3.69	0.124	**0.566**
Female sex, n (%)	32 (78.0%)	14 (56.0%)	0.183	0.482
Employed, n (%)	24 (58.5%)	19 (76.0%)	0.304	0.378
Hypertension, n (%)	24 (58.5%)	7 (28.0%)	0.102	**0.647**
Diabetes mellitus, n (%)	11 (26.8%)	1 (4.0%)	0.105	**0.666**

Values are presented as mean ± SD or number (percentage). Adjusted *p*-values represent comparisons between groups after Holm–Bonferroni correction for multiple testing. Standardized differences were calculated to evaluate baseline comparability between groups. Values above 0.10 were considered indicative of imbalance.

**Table 4:** Clinical characteristics of the patient group.

Variable	Value	Adjusted *p*
Symptom duration (months), median [IQR]	6 [3.75–9]	**0.043**
Radiculopathy, *n* (%)	33 (80.5%)	**<0.001**
NSAIDs use, *n* (%)	27 (65.9%)	**<0.001**
Opioid analgesics, *n* (%)	25 (61.0%)	**<0.001**
MRI performed, *n* (%)	28 (68.3%)	**0.038**
Radiography performed, *n* (%)	20 (48.8%)	0.876

NSAIDs—nonsteroidal anti-inflammatory drugs; MRI—magnetic resonance imaging. Values are presented as median [interquartile range] or number (percentage). *p*-values were adjusted using the Holm–Bonferroni method for multiple testing.

**Table 5:** Cross-sectional NCS parameters of the median nerve in the study groups.

Parameter	Side	Patients	Controls	Effect Size (Cohen’s d/r)	Adjusted *p*
Distal latency (ms)	Right	3.10 [2.78–3.68]	2.10 [1.80–2.53]	0.67	**<0.001**
	Left	3.00 [2.53–3.50]	1.90 [1.60–2.40]	0.74	**<0.001**
Residual latency (ms)	Right	2.13 [1.70–2.72]	0.91 [0.73–1.46]	0.79	**<0.001**
	Left	1.95 [1.68–2.54]	1.10 [0.80–1.33]	0.78	**<0.001**
CMAP amplitude (mV)	Right	5.66 ± 2.49	9.94 ± 2.10	1.83	**<0.001**
	Left	5.79 ± 2.84	9.59 ± 1.84	1.53	**<0.001**
Motor conduction velocity (m/s)	Right	54.30 [48.03–58.25]	58.40 [56.93–59.03]	0.45	**0.002**
	Left	53.10 [47.48–57.78]	58.10 [57.43–59.00]	0.49	**<0.001**

CMAP—compound muscle action potential. Data are presented as mean ± standard deviation or median [interquartile range], depending on distribution. Between-group comparisons were performed using the independent samples *t*-test or Mann–Whitney U test. Effect size is reported as Cohen’s d or r, as appropriate. Adjusted *p*—*p*-value corrected using the Holm–Bonferroni method. A *p*-value < 0.05 was considered statistically significant.

**Table 6:** Cross-sectional semiquantitative sEMG parameters of the APB muscle in the study groups.

Parameter	Side	Patients, Median [IQR]	Controls, Median [IQR]	Effect Size (r)	Adjusted *p*
Amplitude	Right	2 [2–4]	5 [5–5]	0.95	<0.001
	Left	2 [2–4]	5 [5–5]	0.95	<0.001
Recruitment pattern	Right	2 [2–3]	5 [5–5]	0.91	<0.001
	Left	2 [2–3]	5 [5–5]	0.95	<0.001

APB—abductor pollicis brevis; IQR—interquartile range. Data are presented as median [interquartile range]. Between-group comparisons were performed using the Mann–Whitney U test. *p*-values were adjusted using the Holm–Bonferroni method. Effect size r was calculated as |Z|/√N. Statistical significance was set at *p* < 0.05.

**Table 7:** Correlations between ΔVAS and clinical parameters.

Variable	Spearman’s ρ	Adjusted *p*
Symptom duration	0.013	0.936
Age	0.147	0.358
BMI	−0.022	0.891
Hypertension	0.157	0.326
Diabetes mellitus	−0.146	0.363

ρ represents Spearman’s correlation coefficient. Adjusted *p*-values after the Holm–Bonferroni method for multiple comparisons.

**Table 8:** Longitudinal evolution of median nerve conduction parameters after rehabilitation treatment.

Parameter	Side	T0	T1	Effect Size (Cohen’s d/r)	Estimated MCID	Mean Change	Adjusted *p*
Distal latency (ms)	Right	3.10 [2.78–3.68]	2.80 [2.28–3.33]	0.79	0.900 ms	−0.308 ms	**<0.001**
	Left	3.00 [2.53–3.50]	2.70 [2.30–3.28]	0.76	0.488 ms	−0.246 ms	**<0.001**
Residual latency (ms)	Right	2.13 [1.70–2.72]	2.09 [1.61–2.58]	0.86	0.825 ms	−0.185 ms	**<0.001**
	Left	1.95 [1.68–2.54]	1.84 [1.59–2.40]	0.80	0.457 ms	−0.121 ms	**<0.001**
CMAP amplitude (mV)	Right	5.66 ± 2.49	6.17 ± 2.41	0.82	1.225 mV	0.514 mV	**<0.001**
	Left	5.79 ± 2.84	6.03 ± 2.84	0.56	1.422 mV	0.243 mV	**0.004**
Motor conduction velocity (m/s)	Right	54.30 [48.02–58.25]	55.70 [48.40–59.00]	0.69	3.295 m/s	0.614 m/s	**<0.001**
	Left	53.10 [47.47–57.77]	54.10 [48.05–57.60]	0.52	3.414 m/s	0.391 m/s	**0.006**

CMAP—compound muscle action potential. Data are presented as median [interquartile range] for abnormally distributed variables and mean ± standard deviation for normally distributed variables. T0 = baseline; T1 = 6-month follow-up. Effect size was expressed as r for Wilcoxon tests and Cohen’s d for paired *t*-tests. *p*-values were adjusted for multiple comparisons using the Holm–Bonferroni method. Statistical significance was set at *p* < 0.05. Estimated MCID values were calculated using a distribution-based method (0.5 × pooled SD). These values represent exploratory estimates and not validated anchor-based MCID thresholds. Mean change was calculated as T1 minus T0.

**Table 9:** Longitudinal changes in semiquantitative sEMG parameters of the APB muscle after rehabilitation treatment.

Parameter	Side	T0, Median [IQR]	T1, Median [IQR]	Effect Size (r)	Adjusted *p*
Amplitude	Right	2 [2–4]	3 [3–4]	0.75	0.006
	Left	2 [2–4]	4 [3–4]	0.73	0.006
Recruitment pattern	Right	2 [2–3]	3 [3–4]	0.73	0.006
	Left	2 [2–3]	3 [2.75–4]	0.75	0.006

T0 = baseline assessment; T1 = post-treatment assessment. Data are presented as median [interquartile range]. Paired comparisons were performed using the Wilcoxon signed-rank test. Effect size r was calculated for the Wilcoxon test. *p*-values were adjusted for multiple comparisons using the Holm correction. Statistical significance was set at *p* < 0.05.

## Data Availability

The data presented in this study are available upon request from the corresponding author (R.R.O.) and are not publicly available due to privacy restrictions.

## Abbreviations

The following abbreviations are used in this manuscript:

APB	Abductor pollicis brevis
ANCOVA	Analysis of covariance
BMI	Body mass index
CI	Confidence interval
CMAP	Compound muscle action potential
ΔCMAP	Change in CMAP amplitude (T1–T0)
ΔVAS	Change in pain intensity
IQR	Interquartile range
MRI	Magnetic resonance imaging
NCS	Nerve conduction studies
NSAIDs	Nonsteroidal anti-inflammatory drugs
sEMG	Surface electromyography
VAS	Visual Analog Scale
VIF	Variance inflation factor

## Appendix A. STROBE Checklist

The manuscript was prepared and reported in accordance with the Strengthening the Reporting of Observational Studies in Epidemiology (STROBE) Statement [59]. The completed STROBE checklist is provided below to facilitate the assessment of reporting quality and methodological transparency.

**Table A1:** STROBE checklist.

	Item No.	Recommendation	Page No.
Title and abstract	1	(a) Indicate the study’s design with a commonly used term in the title or the abstract	1
		(b) Provide in the abstract an informative and balanced summary of what was done and what was found	1–2
Introduction			
Background/rationale	2	Explain the scientific background and rationale	2
Objectives	3	State specific objectives, including any prespecified hypotheses	2
Methods			
Study design	4	Present key elements of study design	3
Setting	5	Describe the setting, locations, recruitment period, follow-up, and data collection	3
Participants	6	(a) Eligibility criteria and methods of participant selection	3–4
		(b) Matching criteria	Groups were not matched
Variables	7	Define outcomes, predictors, potential confounders, and diagnostic criteria	3
Data sources/measurement	8	Data sources and measurement methods	3–6
Bias	9	Efforts to address potential bias	6
Study size	10	Explain study size	8
Quantitative variables	11	Explain handling of quantitative variables	8–9
Statistical methods	12	(a) Statistical methods	8–9
		(b) Subgroup and interaction analyses	8–9
		(c) Missing data	No missing data
		(d) Loss to follow-up	No loss to follow-up
		(e) Sensitivity analyses	8–9
Results			
Participants	13	(a) Participants at each stage	9–10
		(b) Reasons for non-participation	N/A
		(c) Flow diagram	Not included
Descriptive data	14	(a) Characteristics of participants	9–10
		(b) Missing data	N/A
		(c) Follow-up time	11–13
Outcome data	15	Report numbers of outcome events or summary measures	10–12
Main results	16	(a) Main results (adjusted estimates)	10–13
		(b) Category boundaries	N/A
		(c) Absolute risk	N/A
	17	Other analyses	13
Discussion	18	Summarize key results	13–16
	19	Discuss limitations	15–16
	20	Interpretation	13–16
	21	Generalizability	16
Other information	22	Funding	16

## Appendix B. TIDieR Checklist

The rehabilitation intervention was reported according to the Template for Intervention Description and Replication (TIDieR) checklist [30] and guide to improve intervention transparency and reproducibility.

**Table A2:** TIDieR checklist for the multimodal rehabilitation program.

Item	Description	Study-Specific Information
1. Brief name	Name or phrase describing the intervention	Multimodal rehabilitation program for patients with cervical spondylosis.
2. Why	Rationale, theory or goal	The intervention aimed to reduce pain, improve cervical mobility, enhance neuromuscular control, and restore upper limb function through a combination of balneotherapy, electrotherapy, thermotherapy, and kinetotherapy.
3. Materials	Materials used	Bathtub with carbonated mineral water, TENS and ultrasound device (1 MHz), paraffin–wax applications, treatment bed and exercise equipment.
4. Procedures	Procedures, activities and processes	The rehabilitation program included carbonated mineral water baths, TENS, ultrasound, paraffin applications and a supervised kinetotherapy program consisting of cervical mobility exercises, isometric strengthening, deep cervical flexor training, scapulohumeral stabilization and stretching exercises.
5. Who provided	Provider expertise	The rehabilitation program was prescribed and supervised by a specialist in Physical and Rehabilitation Medicine and executed by physiotherapists.
6. How	Mode of delivery	Face-to-face, individually supervised.
7. Where	Location	Rehabilitation clinic, Timiș County, Romania.
8. When and how much	Number, duration, intensity	Fourteen consecutive daily sessions. CO_2_ baths: 20 min + 1 h rest; TENS: 20 min; ultrasound: 1 MHz, 0.5 W/cm^2^ continuous, ~1 min/5 cm^2^; paraffin: 20 min + 1 h rest; kinetotherapy: approximately 40 min/day.
9. Tailoring	Personalization	Exercise intensity and range of motion were progressively increased according to patient tolerance, particularly in patients with cervical radiculopathy.
10. Modifications	Changes during study	No modifications to the intervention protocol were made during the study.
11. How well (planned)	Adherence/fidelity assessment	All sessions were performed under the supervision of a specialist in Physical and Rehabilitation Medicine. Participants were instructed to continue home exercise twice weekly; however, adherence was not monitored.
12. How well (actual)	Extent intervention delivered as planned	All participants completed the 14-day supervised rehabilitation program. Home exercise adherence during follow-up was not evaluated.
